# Practical guide for marine exo-metabolomic sample preparation

**DOI:** 10.1093/ismejo/wrag115

**Published:** 2026-05-12

**Authors:** Catherine C Bannon, Jana K Geuer, Lennart Stock, Bruna Y P Imai, Manuel Liebeke

**Affiliations:** Department of Symbiosis, Max Planck Institute for Marine Microbiology, Bremen, 28359, Germany; Department of Symbiosis, Max Planck Institute for Marine Microbiology, Bremen, 28359, Germany; MARUM-Center for Marine Environmental Sciences, University of Bremen, Bremen, 28359, Germany; Department of Symbiosis, Max Planck Institute for Marine Microbiology, Bremen, 28359, Germany; Department of Metabolomics, Institute for Human Nutrition and Food Science, University of Kiel, Kiel, 24118, Germany; Department of Symbiosis, Max Planck Institute for Marine Microbiology, Bremen, 28359, Germany; Department of Metabolomics, Institute for Human Nutrition and Food Science, University of Kiel, Kiel, 24118, Germany

**Keywords:** seawater, dissolved organic material, environmental metabolomics

## Abstract

Metabolomic strategies are being increasingly applied in marine systems, leading to unprecedented insights into the ocean and its inhabitants. In particular, exo-metabolites, small molecules produced by marine organisms and released into the environment, have received recognition as factors that influence marine microbial communities, global nutrient cycles, and ecosystem function. Studying exo-metabolites in the marine system is challenging due to the low metabolite concentrations, high salt content, and substantial chemical diversity, all of which can interfere with metabolite detection and therefore necessitate specialized, often tedious sample preparation. Advantageously, new marine metabolomic methods have recently emerged that improve the coverage and detection of key metabolite classes. Here, we offer a practical guide for selecting suitable extraction methods based on specific research needs alongside a systematic review of five recently published methods designed for marine exo-metabolomics. Specifically, these workflows enable quantification of various primary metabolites using liquid and gas chromatography coupled to mass spectrometry. The selection process is guided by several key questions that consider both experimental design and the specific research questions. Additionally, we highlight both the practical constraints and aspects of sample handling for each method. This guide aims to support researchers in effectively choosing a method that aligns with their study goals and logistical capabilities, while also highlighting the opportunities for innovation in the field moving forward.

## Introduction

**Figure 1:**
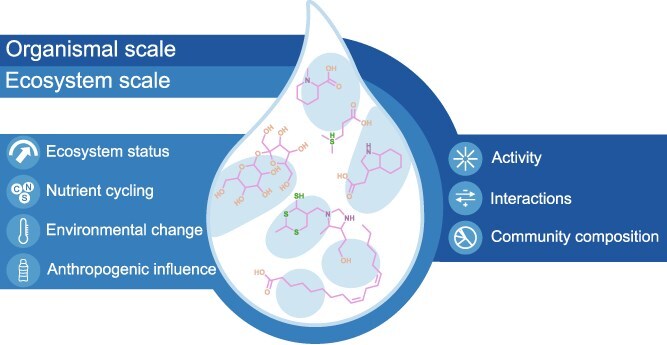
Insights provided by marine exo-metabolomics.

Metabolomics is the study of metabolites produced and used within a biological system. Although lagging behind other omics approaches, metabolomics has been increasingly applied to marine ecosystems, affording unique insights into connections between chemicals, organisms, and environments (Fig. 1). In the ocean, where life unfolds entirely in water, many metabolites are found outside of cells, dissolved directly in the surrounding seawater, a collective pool known as the exo-metabolome.

The exo-metabolome contributes to the broader pool of marine dissolved organic matter (DOM), although individual metabolites typically represent only a small fraction of total DOM composition. DOM is an operational term that is defined as the organic material, which includes dissolved metabolites as well as membrane fragments, virions, and vesicles, that passes through a filter, most commonly ≤0.22 μm. It is widely considered to be one of the most chemically complex mixtures on earth [1, 2]. This pool contains small metabolites that have been released, either passively or actively, by organisms into the seawater as well as those formed through extracellular transformation processes such as enzymatic or abiotic reactions. It spans a wide range of metabolite sizes, polarities, and charges, and is composed of chemical classes such as amino acids, lipids, and alcohols, as well as functionally defined molecules like cofactors. The exo-metabolome can be, and has been, categorized by chemical class, lability, elemental composition, functional class [3], and/or ecological role [4]. Together, this metabolite pool provides unique insights into the ocean on both an organismal and ecosystem scale (Fig. 1). The exo-metabolome encompasses molecules that drive biogeochemical cycles [3, 5, 6] and serve as indicators of ecosystem status [7, 8] and anthropogenic impact [9, 10]. Many exo-metabolome molecules can serve as cues or signals for inter- and intraspecies interactions [11–13], while others can serve as indicators for community composition [14, 15] and microbial metabolic processes [16]. Measuring metabolites in seawater has been central to understanding their roles in these biogeochemical and ecological processes and interpreting the significance of their presence, absence, and abundance. Despite growing interest in marine metabolomics and the insights it provides, measuring the marine exo-metabolome remains a significant challenge.

Analyzing the exo-metabolome in marine samples is challenging because it is a highly diverse and dynamic chemical pool present at extremely low concentrations in a high salt content matrix (Box 1). Specifically, small polar metabolites that represent a significant portion of the exo-metabolome both in concentration and importance are routinely lost throughout common analytical workflows [17, 18]. Widening appeal and the increased access to analytical instruments have led to the development of methods that look to overcome the inherent obstacles of marine exo-metabolomics within the last decade [18–22]. Despite these individual advancements, a structured overview of the most useful approaches has not been systematically performed, making it particularly difficult for those looking to enter the field.

**Box 1**

*How salty is seawater? Why does this matter?* When working with marine samples, scientists face a significant challenge that remains an active area of methodological developments: high salt content. Salt is present in seawater at millimolar concentrations, which is orders of magnitude higher than that of metabolites of interest (nM to pM). On average, seawater contains 35 g/L of dissolved salts, which is about two magnitudes higher than the salt content of urine, another analytically challenging matrix [21]. Beyond their abundance, salts present a major analytical challenge as the chemical properties of both salt and metabolites are similar, like high solubility, ionic nature, and strong interactions with chromatographic systems. Together they interfere with the detection and separation of low-abundance metabolites. Effectively managing this salt background is therefore a key consideration in measuring the marine exo-metabolome.

Here, we provide an accessible guide highlighting sample preparation workflows designed for both targeted and untargeted marine exo-metabolomics. We outline the capabilities and limitations of these methods, specifically tailored for researchers entering the field. We first provide an overview of common workflow steps up to metabolite detection, leading to a set of guiding questions for a marine exo-metabolomic method selection. We systematically compare five methods for the quantitative analysis of marine exo-metabolites in seawater, aiding in the selection of the most suitable approach for one’s research needs. These methods tackle core challenges of marine exo-metabolomics including high salinity, low metabolite concentrations, and chemical diversity using different approaches and have been validated to perform robustly in seawater. Finally, we consider future directions that would benefit the broader field and enable new scientific insights.

## General workflow overview

**Figure 2:**
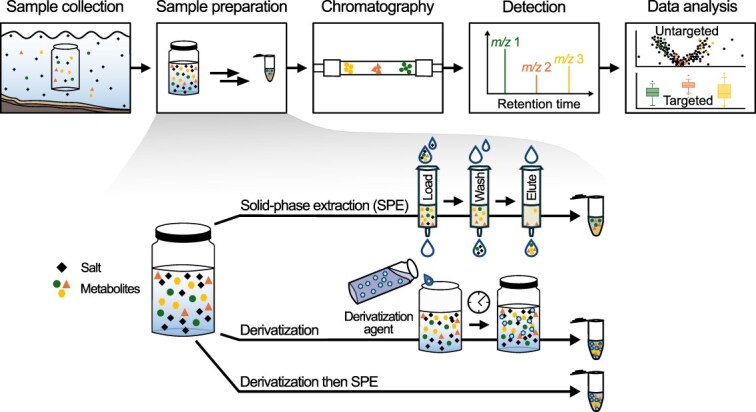
Overview of typical marine exo-metabolomic experiment. Common steps of marine exo-metabolomic experiments include sample collection, sample preparation, chromatography, detection, and data analysis. Sample preparation methods can include solid-phase extraction (SPE) represented by a SPE resin-containing cartridge, derivatization or a combination of both (derivatization then SPE), each resulting in a different sample of extracted metabolites for analysis.

Understanding the general workflow of marine exo-metabolomics protocols, and how they can vary, is critical to evaluate methods and envision their application in the lab and field work successfully. Workflows typically involve five key steps (Fig. 2). Although mass spectrometry (MS) acquisition, data analysis, and data interpretation are essential components of metabolomics, they are well covered in existing literature [23–27]. In this guide, we place emphasis instead on the critical upstream step of sample preparation that influences data quality.

(i) Sample collection: You can collect seawater or experimental samples at various volumes. Pre-filtration using a larger pore size (> 0.7 μm) removes large cells and particles and reduces clogging and mechanical stress during subsequent filtering through a smaller final filter (usually 0.22 μm). The filtrate contains the exo-metabolites and should be processed immediately, for example by acidification and/or storage at −20°C or below, as is common practice in the field, until further analysis. Acidification can influence metabolite stability and recovery; it may negatively affect specific metabolites of interest, for example through the hydrolysis of carbohydrates, whereas improve the retention of others during SPE [22]. Finally, many collection methods also require rigorous cleaning routines and pre-combusted glassware to avoid plastic for the contaminants it introduces into untargeted metabolomics [22].

(ii) Sample preparation: Sample preparation is the process of generating an analytical sample suitable for measurements from the filtrate, typically through workflows that leverage derivatization and/or extraction.

*Derivatization*: Metabolites in the filtered sample can be stabilized and then selected for using various types of derivatizations. Derivatization is a chemical process that modifies specific functional groups of metabolites, thereby altering their chemical properties. These modifications improve their separation from salts, extractability, or detectability in subsequent analyses.

*Extraction*: Metabolites (derivatized or not) can then be extracted, usually using solid-phase extraction (SPE), in order to separate them from the salt that interferes with analytical measurements and to concentrate them (see Box 1). During SPE, a sample passes through a resin-filled cartridge that retains the metabolites of interest based on size, charge, and/or polarity while flushing out salts. You then elute (release) the bound metabolites with a solvent, resulting in a less salty, concentrated sample that is usually dried and then reconstituted before the next steps.

(iii) *Chromatography*: Even when metabolites are concentrated in a salt-free sample, their diversity can make it challenging to detect all of them simultaneously. Chromatography, such as high-performance liquid chromatography (HPLC) and gas chromatography (GC), is commonly used to further separate metabolites based on polarity, size, or other physicochemical properties such as charge state, hydrophobicity, or volatility before detection. Some approaches analyse samples by direct injection without chromatography, which can be advantageous for detection strategies like Fourier-Transform ion cyclotron resonance mass spectrometry (FT-ICR-MS) which can provide broad chemical profiling [17].

(iv) *Detection*: MS has become the leading analytical approach used to detect, identify, and quantify metabolites. After separation, metabolites are ionized (addition of a charge), directed through different sections of the instrument, and “weighed” by a detector yielding a *m/z* (mass over charge). The intensity of the *m/z* correlates with the abundance of that specific ion, although this correlation is not always linear and cannot be compared across *m/z* values [28]. Nuclear magnetic resonance spectroscopy is another common detection technique to characterize metabolites and compare metabolic profiles, offering high reproducibility [29]. To generate reliable data, quality controls such as process blanks, internal standards, repeated pooled sample injections, and randomization of samples during measurement are advised and are expanded on in the supplemental text (Section 2).

You can select for and against metabolites within a sample at each step of the workflow (Fig. 2), influencing all downstream analyses and results. For example, there are many different types of SPE cartridges, some of which are more suited for some metabolites than others. Most commonly, resins based on a modified styrene-divenylbenzene (DVB) polymer are used that have superior retention of medium-sized, slightly polar metabolites. These resins are offered by different brands and are commonly used, such as PPL (Priority PolLutant, Agilent) or HLB (hydrophilic–lipophilic balance, Waters, Chromabond [30, 31]). They are, however, less efficient with highly polar metabolites as these are flushed out with the salts during washing steps [22, 32].

Analytical platforms also have specific limitations [33]. Gas chromatography has a size cut-off (~600 Da, including the derivatization agent) that makes larger metabolites impossible to detect, and triple-quadrupole mass spectrometers are sensitive, but lack the resolution for untargeted techniques. Even if some mass spectrometers can detect ions below *m/z* 100, this lower mass range is rarely analyzed because it is dominated by background ions. Additionally, chromatographic column selection, mobile phase composition and solvent gradient choice can strongly affect detection sensitivity [34]. The variability and consequences of each step make choosing a suitable method for your research a challenging task, especially if you are entering the field.

## Guiding questions

We suggest that clarifying the desired data type and metabolites of interest, together with defining your experimental design, are essential starting points to effectively navigate the decision-making process of selecting a marine exo-metabolome method for your work. Although each presented question is useful, the order of importance of these questions is case-specific. That is to say, you may have a class of metabolites in mind, then search for analytical platforms that work for these, or you might have access to a metabolomics facility and are not tied to certain metabolites or research questions. Ultimately, the answers to these questions should serve as guiding principles, helping to steer your study in the right direction.

### Question 1: What is your research question?

#### Q1.1 Which metabolites do you want to measure?

In discussions about method development for marine exo-metabolites, the first question that typically arises is, “Which metabolites do you want to measure?” The responses to this question generally fall into three categories: (i) a specific metabolite (or metabolites) (ii) a class of metabolites, and (iii) everything (at once).

*Selected metabolite(s):* You may already have a set of metabolites in mind that you are interested in measuring that have been previously identified as key metabolic currencies in similar studies or are based on the pre-established research objectives. This simplifies workflows, because the methodology for each step can be more easily adjusted to select for the detection and quantification of these specific metabolites based on expected concentrations, size, and chemistry. Informing yourself on the metabolite of interest’s functional groups and chemical composition could streamline picking a derivatization strategy or analytical platform. In this case, we suggest sorting through the metabolites validated in the methods presented here to determine suitability (Table S1) or reviewing literature for methods that have already detected these metabolites in seawater.

*Individual metabolite class:* Metabolites in the same metabolite class, like amino acids, lipids or sugars, generally have similar size, polarity, charge, and ionization ability, which makes them easier to measure together, although exceptions exist. For certain groups of metabolites, established analytical methods may already exist that are specifically optimized for those molecules. To measure them you might therefore not rely on the methods presented in this guide. You can, for example extract lipids from seawater using a non-polar solvent [34], and a suite of B-vitamins can be captured using C_18_ SPE [35]. Traditional measurements like those using a ligand exchange resin [36] are also available for metabolite classes such as amino acids, and additional methods for the quantification of specific metabolite groups in seawater are provided in Table S2.

*Everything at once:* This is a difficult measurement goal, because as outlined in Section 3, any exo-metabolomic workflow will significantly bias metabolites retained in your sample. Extracting and detecting an optimal metabolite (retains to SPE cartridge, mM concentration, functional groups amenable to derivatization, readily ionizable) alongside a difficult-to-detect metabolite (weakly retains to SPE cartridge, pM concentration, prone to abiotic degradation, difficult to ionize) is challenging. This leads to the reality that measuring the complete exo-metabolome in a seawater sample is not possible, especially when only pursuing one method. As a result, it is often more practical to focus on a specific subset of metabolites or adjust expectations accordingly.

#### Q1.2 Are you aiming for quantitative (targeted) or qualitative (untargeted) data?

Metabolomics can generally be categorized into two different approaches, targeted and untargeted (or non-targeted) [37], depending on the type of workflow and MS analysis used. Each approach has unique goals and produces different types of data, which in turn constrain the kinds of questions that you can address. We suggest that one of the most relevant things to consider early on during an exo-metabolomic experiment is your desired data output and, as such, the MS approach used.

##### Untargeted metabolomics

In untargeted (or non-targeted) studies, the primary goal is to capture a comprehensive suite of metabolites in a given sample to assess how metabolomes differ between samples and is key for hypothesis generation and discovery [38]. For marine exo-metabolomics, like with many other complex samples, a comprehensive suite of metabolites is unrealistic because the sample has already gone through several processing steps (Fig. 2) during which losses and biases have been introduced. Due to the enormous complexity and uncertainty of metabolites in marine systems, untargeted analyses moreover usually result in a list of unknown features that are unidentifiable in existing metabolite libraries. Such studies usually lead to putative metabolite identification [39–41] and fingerprinting of molecular formulas [41]. The data generated from these experiments requires significant statistical and computational power to isolate peaks, align runs, identify metabolites, and investigate metabolic networks, for which there are valuable resources further outlined in the supplemental text (Section I). Untargeted workflows involving derivatized samples require additional methodological considerations and optimization. For example, MS/MS scans may not always include the derivatization marker whereas the use of isotopically labeled derivatized reagents can increase confidence in metabolite identification [19]. However, confident structural assignment typically follow-up analyses, such as comparison with authentic standards.

##### Targeted metabolomics

Targeted metabolomics is characterized by searching for specific metabolites in your sample and each step of the workflow can be adjusted to optimize the detection of these metabolites. Although targeted approaches enhance sensitivity and selectivity, they are limited by a predetermined list of metabolites, leaving researchers blind to broader metabolic changes. In contrast, this technique allows for the most reliable absolute quantification and, therefore, should be selected if your research question requires such data. Absolute quantification requires your workflow to be validated using authentic standards, including extraction efficiency (or percent recovery, or process effect) and derivatization efficiency, which measures how much of a given metabolite gets derivatized. Determining extraction and derivatization efficiency is generally challenging as it requires standards and/or pre-derivatized standards that are not always commercially available or affordable.

For both targeted and untargeted approaches, it is best to familiarize yourself with matrix effects. Matrix effects refer to the suppression (or rarely, enhancement) of a metabolite’s signal caused by the simultaneous ionization of all the other molecules in the sample. The matrix effect is particularly problematic in marine samples where background could vary significantly depending on salinity, dominant metabolites, or DOM content. Marine samples have significant matrix effects which have to be accounted for by the use of best matched internal standards [42], internal standards [19, 43], matrix-specific calibration curves [44], or response factor corrections [14].

### Question 2: What are your experimental design and sampling constraints?

Constraints such as limited sample volume or instrument availability can dictate the choice of one method over another, making them key considerations to understand before exploring different methods.

#### Q2.1 How much volume can you sample? What metabolite concentration range do you expect?

One of the most pressing challenges in processing marine exo-metabolomic samples is balancing metabolite concentration with limited sample volume. The lower the concentration of the metabolite of interest, the more sample volume is required to reach a sufficient limit of detection and quantification for a given method and analytical instruments (Box 2). In summary, although it may appear straightforward, sufficient metabolite must be present in your sample at the end of your workflow (Fig. 2) to be detected. This is particularly important to consider if you are working with labile metabolites that are taken up or modified quickly by organisms in a given system or are sensitive to abiotic factors, like light or temperature.

**Box 2**

*How low can you go?* A crucial concept in metabolomics is the limit of detection (LOD) and limit of quantification (LOQ). These values can be calculated in different ways, but, as the names suggest, they give a value of the lowest concentration for which the presence of a substance can be confirmed in a sample, LOD, and the lowest concentration that is necessary for quantification with acceptable accuracy and precision, LOQ. Both LOD and LOQ often differ significantly between metabolites in common solvents, their natural matrix, such as seawater, and the analytical instruments used for data acquisition. You should treat reported detection limits in various methods as general guidelines, as the exact values depend on your specific experimental setup and metabolite they apply to. Therefore, it is important to determine these values under your own conditions and report them with your other metabolome results.

The origin of your samples might predetermine the volume available for measurements. Laboratory experiments often allow for more flexibility whereas field work often comes with pre-determined constraints, such as the sampling of sediment pore-water at high resolution or tight water budgets on oceanographic cruises. Another important consideration is the impact of the sampling itself. Prolonged sample handling steps, like filtration, may influence the physiology of cells or chemical composition of the sample, potentially affecting the metabolites present in the surrounding medium [3, 45]. Additional practical limitations may arise such as insufficient replicates, inaccessible equipment or consumables and inadequate storage options or space. If this is the case, it is important to assess the detection limits of the different methods. A common target is to measure metabolites at nanomolar (nM) concentrations or lower, which most of the methods presented below achieve. When sufficient sample volume is available (≥500 mL), picomolar (pM) sensitivity may be achievable, depending on the use of concentration steps such as SPE, the extraction efficiency for the metabolites of interest and the sensitivity of the analytical platform used [19, 22]. Increased sample volume, however, also requires more processing hours in the lab, increases the risk of abiotic degradation and selective extraction, and may exceed the loading capacities of SPE cartridges.

If the sample volume is not predetermined, it may be helpful if you consider the expected concentration range of the metabolites of interest. For targeted approaches, where specific metabolites are known in advance, you should use the expected concentration, method efficiency, and limits of detection and quantification to back-calculate the required sample volume.

#### Q2.2 Which analytical platforms and resources do you have available?

Another important consideration that is often overlooked in method reviews is the accessibility and availability of analytical instruments and the cost and time of sample processing. The majority of methods rely on an HPLC–MS platform for separation and detection of metabolites during the final steps of the workflow (Fig. 2) but there are ongoing efforts to leverage gas chromatography in this space. GC–MS platforms are commonly used in metabolomics and offer robust, selective and reproducible results in combination with databases [45]. Not all mass spectrometers, however, are made the same - some are more suitable for sensitive targeted analysis (e.g., triple-quadrupole MS) whereas others are essential for untargeted (e.g. high-mass resolution (HR) orbitrap MS). Access to expensive analytical instruments is not the only cost associated with these methods; centrifuges for sample cleanup and phase separation, filters for particle removal, and instruments for drying samples should also be considered. SPE cartridges and derivatization agents are an additional expense to the solvents, plastics, and glass consumables that are required to successfully complete various methods reported here. A more consolidated summary of these additional considerations, such as the time required to perform each method, are explored in detail in the supplemental text (Section II). Methods presented here use different combinations of tools and materials, and we have worked to make these differences transparent to support informed decision making.

## Methods specifically designed for analyzing marine exo-metabolomes

**Figure 3:**
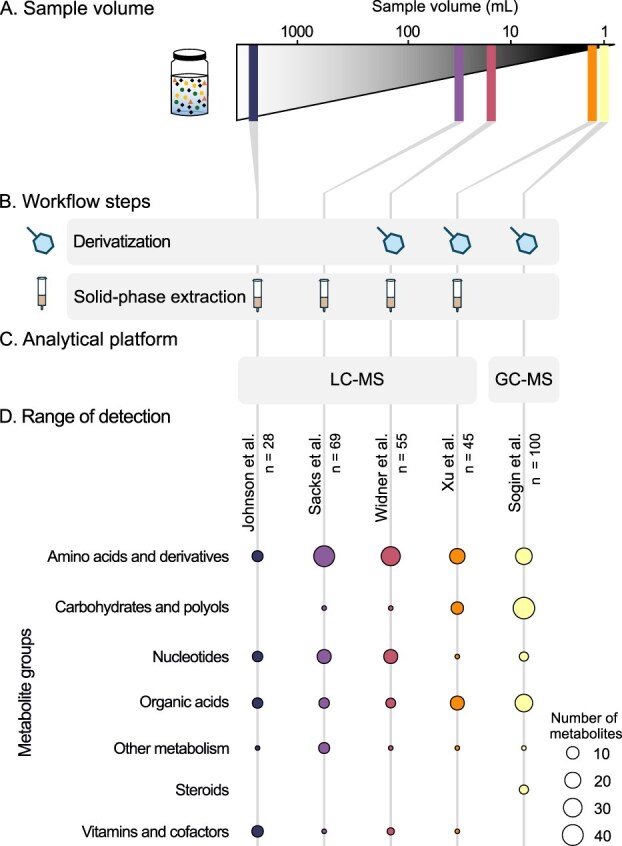
Comparison of marine exo-metabolomic methods. A bubble plot style comparison, showing (A) sample volume, (B) extraction steps, (C) analytical platform used, and (D) range of detection across five marine exo-metabolomics methods reviewed in Section 5. Extended details of protocols are provided in Table 1. Circles represent the number of detectable metabolites in each class, but they may not be the same across each method. The total number of metabolites detected in each method is indicated by n. Metabolite groups were assigned using a semi-automated approach with subsequent author review, although some metabolites may belong to multiple classes. Metabolites included in (D) are reported in Table S1 and a detailed explanation of the production of this figure is provided in the supplemental methods.

Over the past decade, at least five validated and powerful methods have expanded both the detected concentration range and chemical variability of the marine exo-metabolome (Table 1, Fig. 3). These methods have unique sample requirements, metabolite coverage, workflows, and analytical platforms that make choosing the most appropriate method for your research challenging. Each method also has distinct advantages and limitations that should be carefully considered.

**Table 1:** Detailed overview of five marine exo-metabolomic sample preparation methods. LOD = limit of detection.

Method	Johnson *et al.* 2017	Sacks *et al.* 2022	Widner *et al.* 2021	Xu *et al.* 2021 (MetFish)	Sogin *et al.* 2019 (SeaMet)
**Specific area of application**	selected target metabolites	positively charged metabolites	samples with low metabolite concentrations	very salty samples	low volume samples (e.g. porewater)
**Sample volume required**	1000 mL	40 mL	20 mL	1 mL	1 mL
**Range of detection limits**	picomolar to nanomolar	picomolar to nanomolar	picomolar to nanomolar	nanomolar to micromolar	micromolar
**Detected metabolite classes**	low polarity, vitamins, nucleotides, nucleosides, specialized small metabolites	positively charged polar and zwitterionic metabolites, primarily amino acids, nucleotides, nucleosides, amines	alcohol- and amine groups, amino acids, organic acids, nucleotides/nucleosides,	amino acids, organic acids, carbohydrates	amino acids, organic acids, carbohydrates (e.g. sugars, sugar alcohols)
**Specific functional groups targeted**	N/A	N/A	hydroxyl, amine functional groups	amine, carboxylic acid, carbonyl, hydroxyl functional groups	hydroxyl, carboxyl, thiol, amine functional groups
**Sample processing**					
**Preparation steps**	filtration, acidification, SPE	filtration, SPE	filtration, derivatization, SPE	filtration, derivatization, liquid–liquid extraction, in-line SPE	filtration, derivatization
**Derivatization agents**	N/A	N/A	benzoyl chloride	dansylchloride, dansylhydrazine, dansylcadaverine, 4-(dimethylamino)benzoyl chloride	methoxyamine hydrochloride (MeOx) and BSTFA (N,O-bis(trimethylsilyl)tri-fluoroacetamide)
**Solid-phase extraction**	modified styrene-divenylbenzene (DVB) polymer (PPL)	cation-exchange resin (Dowex 50WX8)	modified styrene-divenylbenzene (DVB) polymer (PPL)	N/A	N/A
**Separation**					
**Chromatography**	HPLC	HPLC	HPLC	HPLC	GC
**Analytical column (if applicable)**	reverse phase (C18)	HILIC (pos and neg) or reverse phase (cyano)	reverse phase (C18)	online SPE-nanocapillary LC (C18)	non-polar phenyl arylene polymer (DB-5MS)
**Detection**					
**Method**	RP-HPLC-MS/MS	NP/RP-HPLC-MS/MS	RP-HPLC-MS/MS	HPLC-MS/MS	GC–MS
**Mass spectrometer**	triple quadrupole	orbitrap	orbitrap	orbitrap	single quadrupole
**Approach presented**	targeted, untargeted	targeted, untargeted	targeted	targeted, untargeted	targeted, untargeted
**Targeted quantification approach**	Internal standards spiked into sample post-extraction. Extraction efficiency and LOD provided.	Internal standards used during analysis. Extraction efficiency and LOD provided.	Extraction efficiency (process effect), matrix-matched calibration curves, and LOD provided.	Extraction efficiency and LOD provided.	Calibration with 18 authentic standards, LOD determined for subset based on linear dynamics.
**Studies that have used the method**	[46, 47, 48]		[47, 49]		[50]

Here, we provide a general comparison of these methods, followed by a detailed investigation into each method’s workflow, strengths, and applications. We then highlight additional methods, which may be impactful once validated using analytical standards and environmental samples.

### Johnson *et al*. 2017 (DVB-based SPE)

Johnson *et al*. (2017) built upon a commonly used DOM extraction method by Dittmar *et al*. 2008 [29], by systematically testing the LOD and extraction efficiency of 89 metabolites in different marine matrices. Briefly, 1 L of seawater is collected, filtered, acidified, and then extracted by SPE with DVB-based resin before samples are eluted and dried. In addition, the authors perform matrix-matched calibration curves for all target metabolites to ensure accurate quantification in various marine samples, although few metabolites performed reliably.

This study provided a valuable dataset of metabolite-specific extraction efficiencies and highlights the significant impact that seawater matrices have on the extraction efficiency of small, polar metabolites. Other studies, including Sacks *et al*. (2022) have confirmed and expanded on the quantification of a broad range of metabolites with this method (Table 1). This method, however, requires the largest sample volume of the methods showcased; it is probably susceptible to variations in DOM concentration, and restricted to those few metabolites which are retained on DVB-based SPE.

The SPE method has been the most applied technique so far, and has provided valuable insights into diel cycles and the release of amino acids by sponges [46], the seasonal and diel variability of dissolved metabolites in the northwestern Sargasso Sea [47] and valuable insights into diversity and dynamics of DOM using untargeted methods.

### Sacks *et al*. 2022 (cation exchange SPE)

Sacks *et al*. 2022, used cation-exchange SPE during sample preparation to separate metabolites of interest directly without derivatization from their salty matrix. This approach targets polar zwitterionic metabolites, like amino acids and common osmolytes. The water samples are loaded onto a strong cation exchange resin, then eluted from the SPE cartridge and carefully rinsed with cation exchange cleaned water. After elution into fractions and pH measurement, the basic fractions accounting for ~90% of metabolites of interest are collected for further analysis.

The authors of this method rigorously tested the extraction coverage by running multiple different MS modes (normal phase, reverse phase, HILIC columns) to demonstrate the detection of various metabolites. Evidence from internal standards showed the cation exchange SPE method covered a range of zwitterionic metabolites and the application for untargeted MS was demonstrated. Advantages include that it does not require specific functional groups for derivatization, it is suitable for the quantification of low concentrated metabolites, and is robust to different matrices and DOM concentrations. This method is, however, laborious because it requires packing individual SPE cartridges, preparing cation-exchange cleaned water, and, as suggested by many methods, exclusively using acid-cleaned or combusted material to remove trace organic contaminants. To date, this method has yet to be used in other applications, but it clearly demonstrates the potential for use of exo-metabolomic marine samples.

### Widner *et al.* 2021 (benzoyl chloride derivatization)

Another sensitive and comprehensive HPLC–MS based method was developed by Widner *et al*. (2021). They presented a method that facilitated the sensitive detection of metabolites with amine and/or alcohol functional groups by derivatizing these key metabolites with benzoyl chloride (bc) prior to extraction. Briefly, the protocol includes derivatizing the metabolites directly in 25 mL of filtered seawater, then extracting the metabolites using a DVB-based SPE before analysis on HPLC-MS.

The authors found that the derivatization significantly improved the retention of metabolites during SPE, which allowed for quantification of key metabolites previously missed by DVB-based SPE methods alone. Further advantages of this protocol include a very low detection limit (pM) of a broad range of key metabolites along with the comparatively low sample volume. The laboratory work, however, is more labor-intensive because there are several pH-sensitive derivatization steps and multiple drying steps that require specialized equipment and time, and only metabolites retained on the SPE after derivatization can be measured.

Using this method, researchers were able to detect amines, nucleosides, and sulfonates at a concentration down to pM, and reveal site-specific differences in metabolite profiles of coral reefs with varying amounts of benthic coverage as well as that coastal habitat and hydrodynamics primarily structure exo-metabolite assemblages rather than temporal variability [49, 51].

### Xu *et al*. 2021 (four different derivatizations, MetFISH)

Xu *et al*. (2021) proposed a method called MetFISH, short for **met**abolic **f**luorescence ***i**n **s**itu*  **h**ybridization, which uses derivatization reactions to extract and detect amine, carboxylic acid, carbonyl, or hydroxyl functional groups directly in extremely salty samples. Four different chemicals are used to tag the different functional groups, which allows for downstream enrichment steps that consist of a liquid–liquid extraction, then an inline SPE before detection.

The advantages of MetFISH are its ability to detect metabolites in samples with high salt concentrations (up to 2 M total dissolved salts) while maintaining an nM limit of detection, a linear dynamic range for metabolite concentration up to 5 to 6 orders of magnitude, and high reported reproducibility for the tested molecules. As such, this method is suitable to study extreme systems like brine pools, soda lakes, or hypersaline salt lakes, which are habitats where exo-metabolites have yet to be explored and traditional methods are not suitable to investigate. The untargeted analyses in the study suggest that coverage extends beyond those validated. To target many different functional groups, MetFISH, however, requires chemical complexity with multiple derivatization reactions including cleanup steps. This makes the method labor-intensive, expensive and increases potential variability.

### Sogin *et al.* 2019 (methoximation and trimethylsilylation, SeaMet)

SeaMet is a two-step derivatization process protocol developed by Sogin *et al*. (2019), which relies on derivatization to separate metabolites from salt. The steps of this method include drying small volume samples and the resultant salt pellet with toluene, followed by derivatization with methoxyamine hydrochloride and trimethylsilyl-*N*-methyl trifluoroacetamide that stabilize certain functional groups and increase the volatility of metabolites for GC–MS analysis.

The advantage of this method is the low sample volume requirements (maximum 1 mL) and straightforward workflow that does not remove the salt from the sample. This method has the broadest coverage for functional groups of metabolites, namely amines, sugar alcohols, carboxylic acids, amino acids, sugars, and sterols, which are involved in central metabolism. The limitations, however, include a high LOD (*micro*molar concentrations for most metabolites) and the inherent limitations of metabolite size (<600 Da) that come with the use of GC–MS. The chemical reactions performed during the method are extremely water sensitive, and care should be taken to not have remaining water in samples or reagents for best results.

One application of SeaMet included analysis of hundreds of pore water metabolomic profiles from sediments of seagrass meadows. Among many metabolites detected, the authors quantified sucrose concentrations, which reached up to approximately 1 mM, a concentration 80× higher than previously reported for dissolved total carbohydrate concentrations in marine sediments, providing new insight into dominant metabolites in marine systems [52].

### Summary

The five major methods presented here have significantly expanded our ability to measure metabolites in marine habitats, including nucleosides and nucleotides, fatty acids, amine-, and sulfur-containing metabolites, offering new insights into the type and dynamics of chemical currencies (Fig. 3). For known metabolites these methods highlight a general trend to reduce sample volume, which makes collection, replication, and validation of measurements more feasible than previously. Leveraging derivatization strategies and improving SPE retention is becoming more frequent, and the use and optimization of the SPE method using DVB-based resins is on-going. Research from other labs that have leveraged these methods underscores the importance of method development and exploration in advancing the field. As outlined in the supplemental text (Section III), other methods have also implemented additional techniques, such as particular isocratic gradients, upstream HPLC analysis, or alternative ionization approaches to desalt samples and assess the marine exo-metabolome [53–55].

In general, targeted quantification of exo-metabolites is more straightforward than the analysis of untargeted experiments, which involves complex steps such as metabolite annotation, identification, and validation that continues to be a bottleneck in the field as recently reviewed in detail elsewhere [38, 56]. Although data analysis and interpretation remain outside the scope of this paper, some resources to pursue these approaches are provided in the supplemental discussion. Despite the insights that exo-metabolomic samples can provide, the widespread adoption of such methods into marine studies is limited, and room for innovation is wide.

## Future directions

In general, many goals of the marine exo-metabolomics field align with those of the metabolomic field, including increased reproducibility, raw data sharing, rigorous data reporting (mQACC [57, 58]), and a push toward multi-omics integration to help unravel higher-level interactions and patterns [59, 60]. Certain challenges are, however, unique to the marine system and would benefit from targeted innovation efforts [61].

Metabolomics is the omics technique that is most responsive to environmental influences and, as such, is highly sensitive to time, abiotic fluxes, and environmental changes, unlike the genome of involved organisms. This is particularly concerning with seawater samples that sometimes take hours to reach research vessels from deep sample depths. As recently reviewed, advancing *in situ* sampling methods, like the I-SMEL (*In Situ* Marine moleculE Logger) and non-invasive ways to measure metabolites of interest could provide valuable insights into the ocean’s status [61]. Such strategies, combined with time-series monitoring, offer promising opportunities to identify environmental stressors, a topic of growing interest.

Many marine exo-metabolomic methods have been developed to maximize coverage of core metabolites of microbial metabolism which are expected to play a significant role in biogeochemical fluxes. Innovative ways to capture and analyse a broader range of metabolites could substantially improve characterization of the ocean’s metabolome. For example, identifying specialized molecules can also give important insights into microbial ecology and activity, despite being under-represented in methods presented here (Fig. 3, Table S1).

Extending coverage inherently requires exploring innovative approaches to capture metabolites that are excluded by current workflows. Ion mobility MS is a promising approach, as it adds a layer of structural information for the detected metabolites, helping to distinguish isomers within the mass spectrometer, while having potentially the same retention time [62, 63]. Researchers can find inspiration in other fields, particularly in the development of alternative strategies for chemical derivatization or extraction techniques. The emerging trend of salting out samples has been gaining traction in adjacent fields. In addition, Salting-Out Assisted Liquid–Liquid Extraction or QuEChERS method has been successfully employed for salt samples like urine or those used in pesticide analysis [64, 65]. Implementing these methods in the marine setting will come with challenges, like potentially requiring substantial volumes of solvent, but nonetheless are directions for future exploration.

## Conclusions

Supported by the methods showcased here, marine exo-metabolomic research is gaining traction as a powerful tool to assess the ocean and its inhabitants. By comparing diverse analytical methods, this guide equips researchers to navigate challenges such as high salinity, varying sample complexities, limited sample volume, and instrument availability. From beaches to the deep sea, eavesdropping on microbial communities is becoming more accessible and comprehensive. With future investment in method standardization, data reporting and sharing, and reduced technical, financial, and knowledge barriers for newcomers, the field will continue to grow rapidly.

## Supplementary Material

Supplementary_materials_wrag115

## Data Availability

No original data are produced or analyzed in the presented work.
