# Supplementary material for: Practical guide for marine exo-metabolomic sample preparation
Source: ISME J. 2026 May 12;20(1):wrag115. doi: 10.1093/ismejo/wrag115 (PMC13235718; doi:10.1093/ismejo/wrag115)
Supplement: Supplementary_materials_wrag115 [file supplementary_materials_wrag115.zip › 20260505_Supplemental_Bannon_Clean.docx]

**Practical guide for marine exo-metabolomic sample preparation**Catherine C. Bannon^1^*^#^, Jana K. Geuer^1^*, Lennart Stock^2^, Bruna Y. P. Imai^1,3^, Manuel Liebeke^1,3,#^

**Supplemental Information**

**Document includes the following:**

**Supplemental Discussion**

I) Resources for untargeted approaches in marine exo-metabolomic studies

II) Other considerations; equipment, time, and expenses.

III) Additional marine exo-metabolomic methods of note

**Supplemental Methods**

Preparation of Figure 3.

**Supplemental Tables**

**Table S1: Metabolites validated across marine exo-metabolomic methods.** Metabolite listed are grouped by metabolite class and subclass, and indicate which studies reported validation for each metabolite. A value of “1” denotes validation by the corresponding study. Data is used to generate Figure 3.

**Table S2:** **Targeted techniques for the quantification of specific metabolite classes in seawater.** This list is illustrative, not exhaustive.

**Supplemental Discussion**

**I) Resources for untargeted approaches in marine exo-metabolomic studies**

The chemical diversity of the marine exo-metabolome remains far from fully appreciated, and the potential of untargeted metabolomics in marine systems is similarly underexplored^1,2^. Although the methods focused on in this paper mainly used targeted metabolomics to validate and quantify the protocols outlined in Figure 3 and Table 1, they hold strong potential for untargeted applications. Resources for the implementation of untargeted metabolomics are becoming more widely available to the field. To encourage broader adoption and integration of untargeted approaches, we briefly outline the workflow and highlight resources available to support such efforts.

Untargeted mass spectrometry relies on robust data acquisition like outlined and optimized for marine samples in another work^3^. Obtaining data, however, is just the first step in a lengthy data analysis pipeline. Raw instrument data must first be pre-processed before analysis by converting raw vendor files produced by the mass spectrometer to open formats (.mzML) with tools like MSConvert. Peaks need to be detected, deconvoluted, aligned, normalized, and filtered in samples to reliably identify potential metabolite signals. Certain software tools, both proprietary and open-source, are available for feature extraction and annotation like MZmine4^4^, XCMS^5^, MetaboAnalyst^6^ and MS-DIAL^7^.

Only once peak processing has been performed, you can attempt to identify different features in your sample using tools like SIRIUS^8^ as well as databases including METLIN^9^, MassBank^10^, and GNPS^11^. However, different levels of identification confidence must be considered when working with this type of data^12^. These advances, in parallel with the increasing community effort to share data in public repositories, such as MASST^13^ and MetaboLights^14^, have accelerated identification of unknowns and supported novel discoveries in various fields including in marine systems. Notably, a database dedicated explicitly to marine exo-metabolomics has yet to be consolidated but may be of benefit for future consideration. Finally, untargeted analysis requires advanced statistical analysis to infer any differences and interpret data like explained elsewhere^15,16^. Depending on your research question, you may also be interested in pathway mapping, enrichment analysis or network analysis that add additional analysis of which there are examples elsewhere and are outside the scope of this method guide.

**II) Other considerations; needed equipment, time, quality control, and expenses.**

As always, there are additional constraints that must be considered before selecting an ideal marine exo-metabolomic method, particularly for those entering the field for the first time. Accounting for such practical realities like equipment availability, processing time and cost of workflows is an important consideration for many scientists and could ultimately dictate the most suitable method. Carefully planning around these logistics ensures that method selection is feasible and can be successfully implemented. As such, you should familiarize yourself with the additional equipment usually required for different steps of marine exo-metabolomic methods, then go on to highlight the time and cost of methods discussed in this guide.

***Equipment***: Equipment required for marine exo-metabolomic methods extends beyond central, expensive analytical platforms such as mass spectrometers and chromatographic instruments. For example, all of the methods highlighted in this review require reliable drying equipment, like nitrogen evaporators or vacufuges, for various steps of protocol. Sample filtration equipment consisting of syringes or vacuum filtration units are essential to successfully separate dissolved metabolites from cells and particles. The choice between these two drying techniques usually comes down to sample volume, solvent compatibility, and how many samples are in a batch. Methods relying on solid-phase extraction (SPE) also require a dedicated manifold, either vacuum or pump based, to facilitate extractions, and a low-cost, reliable alternative has yet to be developed and adopted by the field. Other common laboratory equipment for workflows includes a benchtop centrifuge, temperature-controlled orbital shaker, ultrasonic bath, vortex mixer, and pH meters or indicator strips. Most marine exo-metabolomic methods also require ultrapure water and cold storage for samples and extracts, equipment that many scientists may take for granted but often is missing in field experiment settings.

***Time***: Sample preparation is by far the most time-consuming step in most marine exo-metabolomic workflows, including the methods presented here. Filtering samples is a critical and time intensive step that requires patience. Importantly, it must be done to remove particulate organic matter while minimizing the release of intracellular metabolites as well as biotic and abiotic effects on the sample. The total time required to complete each method largely depends on your number of samples, and the experience with the respective workflow. In general, we estimate that all methods presented require a minimum 1-2 full working days for full sample preparation. In most cases, methods that require derivatization include lengthy incubation steps to ensure complete reaction and stabilization of associated metabolites. In addition to such incubations, Sogin et al, 2019 requires an 8-hour drying step that can be completed overnight. After successful derivatization, most methods rely on further cleanup steps, which add to the total sample processing time. Workflows requiring solid phase extraction are usually more constrained by the number of samples that can be extracted at the same time in a manifold, the maximum loading and elution speed (usually between 0.5-5 mL per min^17^) and drying steps after the extraction. In summary, the methods described here require an estimated time between a few hours to a couple of days from sample collection to before data analysis (hours = Johnson and Sacks, days = Sogin, Widner, Xu).

***Quality controls***: Successful marine exo-metabolomic methods require a well-thought-out quality control (QC) strategy that monitors both sample processing and analytical instrument performance. Suggested QC requirements include sample preparation blanks to detect contamination, internal standards to correct for both human and instrument variability and randomization of samples during measurement to minimize bias. In addition, isotopically labelled standards are commonly used to validate critical steps such as derivatization reactions and to assess extraction efficiency, as performed by Widner et al. 2021.

***Expenses***: The most significant expenses associated with marine exo-metabolomics method are from the filters, SPE cartridge, derivatization agents and high-purity organic solvents required for extraction and elution. We would highly recommend beginning by assessing the cost of filtration and SPE materials and/or derivatization reagents used in a potential workflow. SPE cartridges are available at various different sizes and resin types, and are generally one-time use to minimize contamination or carry over. The cation exchange cartridges described in Sacks et al, 2022 can be regenerated for multiple uses, reducing costs. However, regeneration increases the time required to complete the method. Derivatization agents are often cost intensive per sample and require careful attention to ensure stability over time. This multiplies for e.g. the MetFish protocol, presented by Xu et al, 2021, because it requires four independent derivatization agents. Another cost intensive factor for targeted methods is the inclusion of isotope labeled internal standards or labelled derivatization agent, which can significantly increase cost per sample. It should be taken into account that saline samples pose practical challenges which many laboratories and commercial service providers may not routinely handle, and as such should be emphasized when outsourcing such analyses.

I**II) Additional methods of note**

*Zabalegui et al. 2020 (seaomics)*^18^
 A method called ‘seaomics’ specifically designed to distinguish the sea surface microlayer from the underlying water was developed by Zabalegui et al. After freezing, samples are freeze dried for two days, re-dissolved in acetonitrile, and centrifuged. The seaomics method uses a specific ion source (plasma-based Direct Analysis in Real Time Simplified Voltage and Pressure), which allows for direct injection onto the mass spectrometer. While sample preparation is comparatively simple, many optimization steps regarding the ion source and solvent type, and volume are included in this method. Seaomics is non-targeted and not quantitative, but tentative molecule identification can be based on MS/MS experiments, and multivariate statistics enable the determination of differences in water masses.
 Because the ion source used in this method is less affected by high salt levels, the method requires no desalination process, which could potentially lead to sample biases. With this method, general substance classes like fatty alcohols, halogenated compounds, and oxygenated boron-containing compounds could be identified. No further identification of single compounds was, however, included. The data processing method following data acquisition was successfully further developed to detect additional features in the same data set^19^.

*Pontrelli and Sauer, 2021*^20^
 A method designed to analyze exo-metabolites from algal cultures was developed by Pontrelli and Sauer, which only requires centrifugation during sample preparation. The sample is injected onto a specialized column with a constant solvent to desalt exo-metabolites from their matrix before metabolite detection using high resolution mass spectrometry. The salty counter ions are only weakly retained by the solid phase and elute at specific times early in the chromatography. This enables the quantification of certain target metabolites at later retention times.

Ionization effects of the salts on coeluting compounds are mitigated by the distinct elution times of the most relevant counterions. This method can measure several important metabolites including various amino acids, lactate, glyoxylate, shikimate, butyrate, and pyruvate. An advantage of this method is the low sample preparation effort and retention times show high reproducibility. The method performs best when samples are highly concentrated, target compounds are defined, and salt levels are consistent. Salt concentration-dependent ion suppression is, however, unavoidable in this technique, which implies that the presence of salt can modify the peak area of compounds of interest.

The method was further used to study metabolite interactions involved in cross-feeding, to identify specific metabolites that individual species produce and consume^21^. It was furthermore used to examine how environmental metabolites interact with cyanobacterial metabolism^22^.

*Lechtenfeld et al, 2024*^23^
 A method for the direct measurement of DOM with FT-ICR-MS splits the salts from compounds of interest by early elution on an HPLC. The method, developed to tackle the matrix-effects during DVB based solid-phase extraction, aims at increasing the analytical window of conventional analysis DOM analysis and was validated on natural seawater samples. The samples can be injected directly after filtration or stored frozen before analysis however, acidification could be used as a storage alternative. HPLC is coupled to FT-ICR-MS using a large chromatographic column and an extended constant solvent elution step after injection. Furthermore, a post column counter gradient is implemented. This leads to a low interaction between the analytical column and salts in the beginning of the chromatography and a delayed elution of DOM. Even though the salty matrix still influences the most polar molecules in early retention times, many polar compounds which would be lost during DVB based solid phase extraction due to the selectivity of the resin can be detected. As a method aiming for a more qualitative approach to characterize polar compounds, it is untargeted.

Albeit the method was developed for global DOM investigation, the simplification of sample preparation yields opportunities to investigate exo-metabolomes in salty samples and has benefits for investigating biological processes and carbon cycling, especially in areas where the amount of sample is a limiting factor, because the method is predicted to work with very low quantities. So far, the method has been primarily applied in the characterization of DOM, but could be beneficial for the use of environmental metabolomic water analyses^24^.

**Supplemental references**

1. Thukral, M., Allen, A. E., Petras, D. Progress and challenges in exploring aquatic microbial communities using non-targeted metabolomics. *ISME J.* 2023; 17, 2147–2159.
10.1038/s41396-023-01532-8

2. Mauduit, M., Greff, S., Derrien, M., et al. Describing the complex chemistry of benthic seawater: from exometabolite sampling strategies to MS-based metabolomics. *Nat. Prod. Rep.* 2025; 42, 1020–1036.
10.1039/D4NP00064A.

3. Stincone, P., Pakkir Shah, A. K., Schmid, R., et al. Evaluation of data-dependent MS/MS acquisition parameters for non-targeted metabolomics and molecular networking of environmental samples: focus on the Q Exactive platform. *Anal. Chem.* 2023; 95, 12673–12682. 10.1021/acs.analchem.3c01202

4. Heuckeroth, S., Damiani, T., Smirnov, A., et al*.* Reproducible mass spectrometry data processing and compound annotation in MZmine 3. *Nat. Protoc.* 2024; 19, 2597–2641.
10.1038/s41596-024-00996-y

5. Smith, C. A., Want, E. J., O’Maille, G., et al. XCMS: processing mass spectrometry data for metabolite profiling using nonlinear peak alignment, matching, and identification. *Anal. Chem.* 2006; 78, 779–787. 10.1021/ac051437y

6. Pang, Z., Chong, J., Zhou, G., et al. MetaboAnalyst 5.0: narrowing the gap between raw spectra and functional insights. *Nucleic Acids Res.* 2021; 49, W388–W396.
10.1093/nar/gkab382

7. Tsugawa, H., Cajka, T., Kind, T., et al. MS-DIAL: data-independent MS/MS deconvolution for comprehensive metabolome analysis. *Nat. Methods* 2015; 12, 523–526.
10.1038/nmeth.3393

8. Dührkop, K., Fleischauer, M., Ludwig, M., et al. SIRIUS 4: turning tandem mass spectra into metabolite structure information. *Nat. Methods* 2019; 16, 299–302. [10.1038/s41592-019-0344-8](https://doi.org/10.1038/s41592-019-0344-8)

9. Guijas, C., Montenegro‑Burke, J. R., Domingo‑Almenara, X., et al. METLIN: a technology platform for identifying knowns and unknowns. *Anal. Chem.* 2018; 90, 3156–3164.
10.1021/acs.analchem.7b04424

10. Horai, H., Arita, M., Kanaya, S., et al. MassBank: a public repository for sharing mass spectral data for life sciences. *J. Mass Spectrom.* 2010; 45, 703–714.
10.1002/jms.1777

11. Schmid, R., Petras, D., Nothias, L.F., et al. Ion identity molecular networking for mass spectrometry-based metabolomics in the GNPS environment. *Nat. Commun.* 2021; 12, 3832.
10.1038/s41467-021-23953-9

12. Schymanski, E. L., Jeon, J., Gulde, R., et al. Identifying small molecules via high resolution mass spectrometry: communicating confidence. *Environ. Sci. Technol.* 2014; 48, 2097–2098.
10.1021/es5002105

13. Wang, M., Jarmusch, A. K., Vargas, F., et al. Mass spectrometry searches using MASST. *Nat. Biotechnol.* 2020; 38, 23–26.
10.1038/s41587-019-0375-9

14 Yürekten, O., Payne, T., Tejera, N., et al. MetaboLights: open data repository for metabolomics. *Nucleic Acids Res.* 2023; 51, D640–D646.
[10.1093/nar/gkad1045](https://doi.org/10.1093/nar/gkad1045)

15. Pakkir Shah, A. K., Walter, A., Ottosson, F., et al. Statistical analysis of feature-based molecular networking results from non-targeted metabolomics data. *Nat. Protoc.* 2025; 20, 92–162.
10.1038/s41596-024-01046-3

16. Worley, B., Powers, R. Multivariate analysis in metabolomics. *Curr. Metabolomics* 2012; 1, 92–107.
[10.2174/2213235X11301010092](https://doi.org/10.2174/2213235x11301010092)

17. Li, Y., Harir, M., Lucio, M., et al. Proposed guidelines for solid phase extraction of Suwannee River dissolved organic matter. *Anal. Chem.* 2016; 88, 6680–6688; [10.1021/acs.analchem.5b04501](https://doi.org/10.1021/acs.analchem.5b04501)

18. Zabalegui, N., Manzi, M., Depoorter, A., et al. Seawater analysis by ambient mass-spectrometry-based seaomics. *Atmospheric Chem. Phys.* 2020; 20, 6243–6257.
10.5194/acp-20-6243-2020

19. Bueschl, C., Riquelme, G., Zabalegui, N., et al. Tidy-direct-to-MS: an open-source data-processing pipeline for direct mass spectrometry-based metabolomics experiments. *J. Proteome Res.* 2024; 23, 3208–3216. [10.1021/acs.jproteome.3c00784](https://doi.org/10.1021/acs.jproteome.3c00784)

20. Pontrelli, S., Sauer, U. Salt-tolerant metabolomics for exometabolomic measurements of marine bacterial isolates. *Anal. Chem.* 2021; 93, 7164–7171. [10.1021/acs.analchem.0c04795](https://doi.org/10.1021/acs.analchem.0c04795)

21. Pontrelli, S., Szabo, R., Pollak, S., et al. Metabolic cross-feeding structures the assembly of polysaccharide degrading communities. *Sci. Adv.* 2022; 8, eabk3076.
[10.1126/sciadv.abk3076](https://doi.org/10.1126/sciadv.abk3076)

22. Haavisto, V., Landry, Z., Pontrelli, S. High-throughput profiling of metabolic responses to exogenous nutrients in *Synechocystis* sp. PCC 6803. *mSystems* 2024; 9, e00227-24.
[10.1128/msystems.00227-24](https://doi.org/10.1128/msystems.00227-24)

23. Lechtenfeld, O. J., Kaesler, J., Jennings, E. K., et al. Direct Analysis of Marine Dissolved Organic Matter Using LC-FT-ICR MS. *Environ. Sci. Technol.* 2024; 58, 4637–4647.
[10.1021/acs.est.3c07219](https://doi.org/10.1021/acs.est.3c07219)

24. Cochran, D., Powers, R. Fourier Transform Ion Cyclotron Resonance Mass Spectrometry Applications for Metabolomics. *Biomedicines* 2024; 12, 1786. [10.3390/biomedicines12081786](https://doi.org/10.3390/biomedicines12081786)

**Supplemental methods**

Data used for the production of Figure 3 is provided in Table S1 and was collected from the following:

Xu et al, 2021: Supplemental Table 3

- All metabolites were included in Figure 3 and Table S1 of this paper.

Sacks et al, 2022: Table 2

- All metabolites were included in Figure 3 and Table S1 of this paper.

Widner et al, 2021: Table 1

- Metabolites listed as NaN (which indicated that the detection limit could not be calculated due to an inadequate standard curve (R2 < 0.9) in the BATS samples were excluded from Figure 3 and Table S1 of this paper.
Johnson et al, 2017: Table 2

- Metabolites listed a ‘-’ (indicated cases where LOD could not be calculated due to high variablity in the data) or ‘n.r.’ (indicating metabolites were not retained on PPL polymer) were excluded from Figure 3 and Table S1 of this paper.
Sogin et al, 2019: Supplemental Table 1.

- Metabolites reported as “not detected”, without a reliable quant ion, and cysteine and Gly-GLu were excluded. Metabolites listed twice in the table (theorine, methionine, leucine) were recorded once and metabolites with both L and D isomers were recorded once.

Extraction efficiency (or process effect) and limit of detection were listed for all metabolites used in the figure Sacks et al, 2022, Xu et al, 2021, Johnson et al, 2017 and Widner et al 2021. Sogin et al, 2019 confirmed the detection of all metabolites used in Figure 3 using commercial standards, but metabolite recovery was determined for 9 standards (Figure S4, Sogin et al, 2019) and dynamic range of detection was provided for select metabolites (n = 18) in (Supplemental Table 2, Sogin et al, 2019). ChatGPT was used to categorize detected metabolites into KEGG classes and sub-classes, which was then manually confirmed by the authors.

*Copyright usage:*

Xu et al, 2021: This is a work of the U.S. Government and is not subject to copyright protection in the United States. Foreign copyrights may apply.

Widener et al, 2021: Attribution-NonCommercial-NoDerivatives 4.0 International

Johnson et al, 2017: The Creative Commons Attribution License

Sacks et al, 2022: This is an open access article under the terms of the Creative
Commons Attribution-NonCommercial-NoDerivs License, which permits use and distribution in any medium, provided the original work is properly cited, the use is non-commercial and no modifications or adaptations are made.

Sogin et al, 2019: Data are adapted under the terms of Creative Commons Attribution 4.0 International license.
